# Bioactivity-Guided Extract Optimization of *Osmanthus fragrans* var. *aurantiacus* Leaves and Anti-Inflammatory Activities of Phillyrin

**DOI:** 10.3390/plants10081545

**Published:** 2021-07-28

**Authors:** Hwa-Young Song, Da-Eun Jeong, Mina Lee

**Affiliations:** 1 College of Pharmacy and Research Institute of Life and Pharmaceutical Sciences, Sunchon National University, 255 Jungangno, Suncheon 57922, Korea; blueocean33@s.scnu.ac.kr (H.-Y.S.); dmsghktn333@naver.com (D.-E.J.); 2 Institute of Jinan Red Ginseng, 41 Hongsamhanbangno, Jinan-Gun 55442, Korea

**Keywords:** *Osmanthus fragrans* var. *aurantiacus*, phillyrin, RAW 264.7 cells, antioxidant, optimal extraction, anti-inflammation, cytokine

## Abstract

The aim of this study was to identify the optimal extraction conditions for leaves of *Osmanthus fragrans* var. *aurantiacus*. Inhibitory effects of various extracts on NO production were compared. Antioxidant evaluations for total phenol and flavonoid contents were carried out using various extracts of *O. fragrans* var. *aurantiacus* leaves obtained under optimal extraction conditions that showed the greatest effect on NO production. The optimal method for extracting *O. fragrans* var. *aurantiacus* leaves resulted in an extract named OP OFLE. OP OFLE showed DPPH and ABTS radical scavenging activities in a concentration-dependent manner. Phillyrin (PH) was isolated as a major compound from OP OFLE by HPLC/DAD analysis. OP OFLE and PH reduced inducible nitric oxide (iNOS) and cyclooxygenase (COX)-2 protein expression and downregulated proinflammatory cytokines such as interleukin (IL)-1β, IL-6, IL-8, and tumor necrosis factor (TNF)-α in LPS-stimulated RAW 264.7 and HT-29 cells. To determine the signal pathway involved in the inhibition of NO production, a Western blot analysis was performed. Results showed that OP OFLE decreased phosphorylation of extracellular regulated kinase (pERK) 1/2 and the expression of nuclear factor-kappa B (NF-κB). Our results suggest that extracts of *O. fragrans* var. *aurantiacus* leaves and its major components have biological activities such as antioxidative and anti-inflammatory properties.

## 1. Introduction

Inflammation is necessary for the human body’s defensive response to injury, lipid peroxide, and infection. However, prolonged inflammatory processes can exacerbate existing diseases or lead to various new diseases [1]. Various risks factors, including diabetes and high blood pressure, are increased by the injurious effects of low density lipoprotein cholesterol, initiating a chronic inflammatory reaction and resulting in vulnerable plaques, rupture, and thrombosis, and inflammation generally increases the incidence of neurodegenerative diseases, cardiovascular diseases, obesity, and cancer [2]. Inflammation can also induce acute and chronic inflammatory responses in the brain, heart, intestine, kidney, liver, lung, pancreas, and the reproductive system, potentially causing tissue impairment or disease. Both infectious and non-infectious agents and cell damage can activate inflammatory cells and trigger inflammatory signaling pathways [3].

Lipopolyssacharide (LPS)-stimulated macrophages have generally been used for evaluating the anti-inflammatory effects of various materials [4]. LPS can also activate Toll-like receptors (TLR) to induce phosphorylation and activation of MAP kinases (MAPKs) that are important for inducible nitric oxide synthase (iNOS) [5]. Activated macrophages can transcriptionally express iNOS, which can catalyze oxidative deamination of L-arginine to produce nitric oxide (NO) [6]. Once activated by LPS stimulation, macrophages play a decisive role in the inflammatory response by releasing various factors such as NO, prostaglandin mediators, and proinflammatory cytokines such as tumor necrosis factor (TNF)-α, interleukin (IL)-1β, and IL-6 [7]. In addition, under inflammatory conditions, macrophages can significantly increase the production of both NO and superoxide anion (O2), which can rapidly react with each other to form peroxynitrite anion (ONOO), thus activating COX-1 and COX-2 as constitutive and inducible forms of cyclooxygenase, respectively [8]. Activation of nuclear factor-κB (NF-κB), an important transcription factor that regulates the transcription of TNF-a, IL-1β, and genes encoding inducible enzymes such as COX-2 and iNOS, is involved in immune and inflammatory responses [9]. Among mitogen-activated protein kinases (MAPKs), extracellular signal-regulated kinase (ERK) and p38 MAPK are thought to play a key role in the regulation of pro-inflammatory cytokines for cellular responses [10]. Intestinal epithelium is a crucial gut mucosal barrier that participates in innate immunity [11]. The IL-8 production can be induced by other inflammatory signals such as LPS, IL-1, and TNF [12].

Natural antioxidants are of increasing interest as medicines and food additives [13]. The use of antioxidants to prevent oxidative damage caused by excessive free radicals is significant for the cosmetics, medical, and food industries, among others [14]. Oxidative stress is associated with cancer, Alzheimer’s disease, diabetes, aging, obesity, Parkinson’s disease, inflammation, and other diseases [15]. Reactive oxygen species can induce protein oxidation, lipid oxidation, base modification, DNA strand break, and modulation of gene expression [16].

The *Osmanthus fragrans* var. *aurantiacus*, as an evergreen tree, belongs to the Oleaceae family. It is a rich source of iridoid, secoiridoid, phenylpropanoid, and lignan glycosides [17]. In a previous study, we determined the anti-inflammatory activities of fractions and triterpenoids from *O. fragrans* var. *aurantiacus* leaves [18]. Natural products are affected not only by the species and tissues used for extraction, but also by the extraction process such as temperature, pretreatment time, and extraction time [19]. The purpose of this study was to discover phytochemicals with anti-inflammatory activities in *O. fragrans* var. *aurantiacus* leaves extract (OFLE) using optimal extraction methods. Various extraction conditions were tested to find the optimal one for preparing an extract with potent anti-inflammatory properties. To determine the antioxidant activity of OFLE, radical scavenging activity was assessed using 1,1-diphenyl-2-picrylhydrazyl (DPPH) and 2,2-azino-bis-30ethylbenzthiazoline-6-sulphonic acid (ABTS) assays. In addition, total phenolic acid and flavonoid contents in the OP OFLE were analyzed. Finally, the anti-inflammatory activities of OP OFLE and its major components were evaluated using LPS-stimulated RAW 264.7 and HT-29 cells.

## 2. Results

### 2.1. Effects of OFLEs Extracted at Various Solvent Ratios on LPS-Induced NO Production and Cytotoxicity

**Figure 1:**
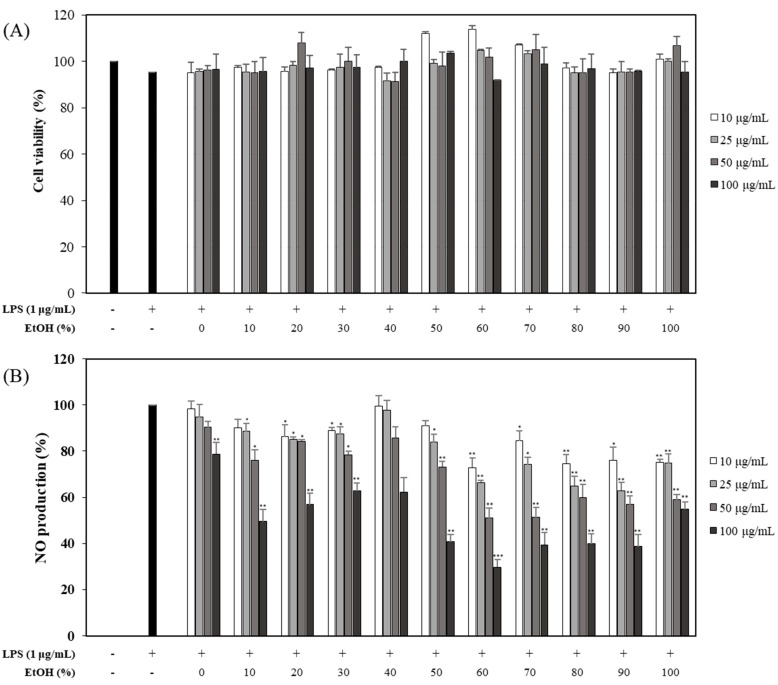
Effects of OFLEs obtained with various solvent ratios on cell viability and NO production. RAW 264.7 cells were cultured in the presence of eleven OFLEs (harvested in June) at various concentrations (10, 25, 50, and 100 μg/mL) for 1 h and then induced with LPS (1 μg/mL) for 20 h. Cell viability (**A**) and NO production (**B**) were measured using MTT assay and Griess reagent, respectively. Nitrite concentrations of non-treated and LPS-treated controls were 0.9 ± 0.1 μM and 17.6 ± 0.2 μM, respectively. Each experiment was performed in triplicates. Data are presented as mean ± SD. * *p* < 0.05, ** *p* < 0.01, *** *p* < 0.001 vs. LPS-treated group.

RAW 264.7 cells were pretreated with eleven OFLEs (extracted with 0, 10, 20, 30, 40, 50, 60, 70, 80, 90, and 100% EtOH for leaves harvested in June) at various concentrations (10, 25, 50, and 100 μg/mL) for 1 h and then induced with LPS (1 μg/mL) for 20 h. The control group was not treated with either LPS or OFLEs. The supernatant (100 μL) was harvested and then NO production was measured with a Griess reagent. Viabilities of cells treated with the OFLEs were measured by MTT assay. Effects of eleven OFLEs (0, 10, 20, 30, 40, 50, 60, 70, 80, 90, 100% EtOH extracts) on NO production and cell viability were then analyzed. Results showed that all OFLEs inhibited LPS-induced NO production in a concentration-dependent manner. They showed no cytotoxicity (Figure 1A). Such inhibitory effects of OFLEs on NO production were not attributable to their effects on cell viability. Inhibitory activities of OFLEs extracted with 60, 70, and 80% EtOH on NO production (71.3, 61.6, and 41.1% inhibition, respectively) were significantly higher than those of other OFLEs at a concentration of 100 μg/mL (Figure 1B).

### 2.2. Effects of OFLEs Extracted with Various Solvent Ratios for Leaves Harvested at Different Time on LPS-Induced NO Production and Cell Viability

**Figure 2:**
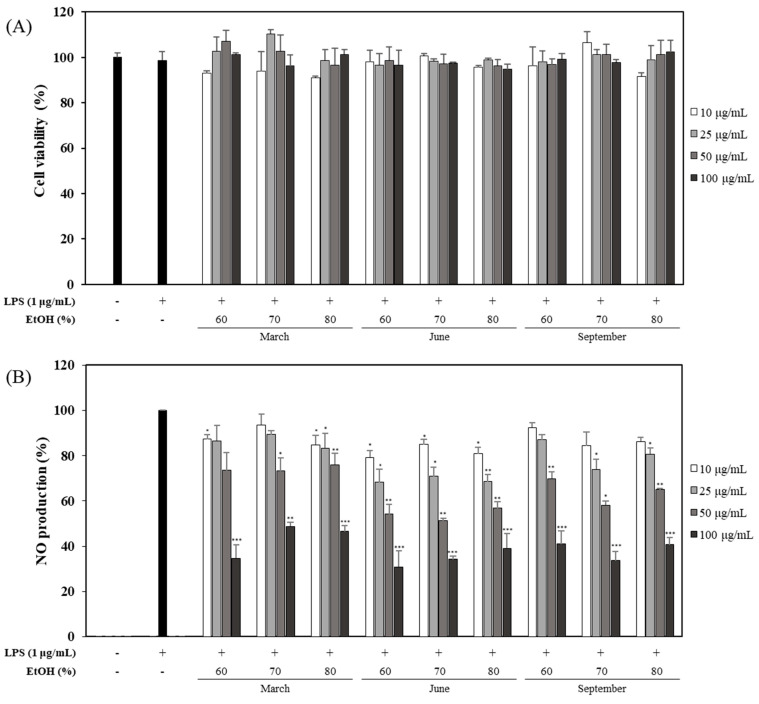
Effects of OFLEs obtained from leaves with different harvest time and various solvent ratios on cell viability and NO production. Inhibitory effects of different harvest time OFLE (10, 25, 50, and 100 μg/mL) by solvent ratio in LPS-induced RAW 264.7 cells. Cells were treated with each extract for 1 h and then stimulated with LPS (1 μg/mL) for 20 h. Cytotoxicity (**A**) was determined by MTT assay. Culture media were evaluated for NO production (**B**) using Griess reagent. Each determination was conducted in triplicates. Data are represented as mean ± SD. * *p* < 0.05, ** *p* < 0.01, *** *p* < 0.001 vs. LPS-treated group.

After the first experiment, nine OFLEs (extracted with 60, 70, and 80% EtOH for leaves harvested in March, June, and September) at different concentrations were also evaluated for their effects on NO production and cell viability. Inhibitory effects of OFLEs on NO production were not attributable to their effects on cell viability (Figure 2A). For leaves harvested in June, the OFLE extracted with 60% EtOH showed a significantly higher inhibitory activity (69.1% inhibition) on NO production than other OFLEs at a concentration of 100 μg/mL (Figure 2B).

### 2.3. Effects of OFLEs Obtained with Various Extraction Time on LPS-Induced NO Production and Cell Viability

**Figure 3:**
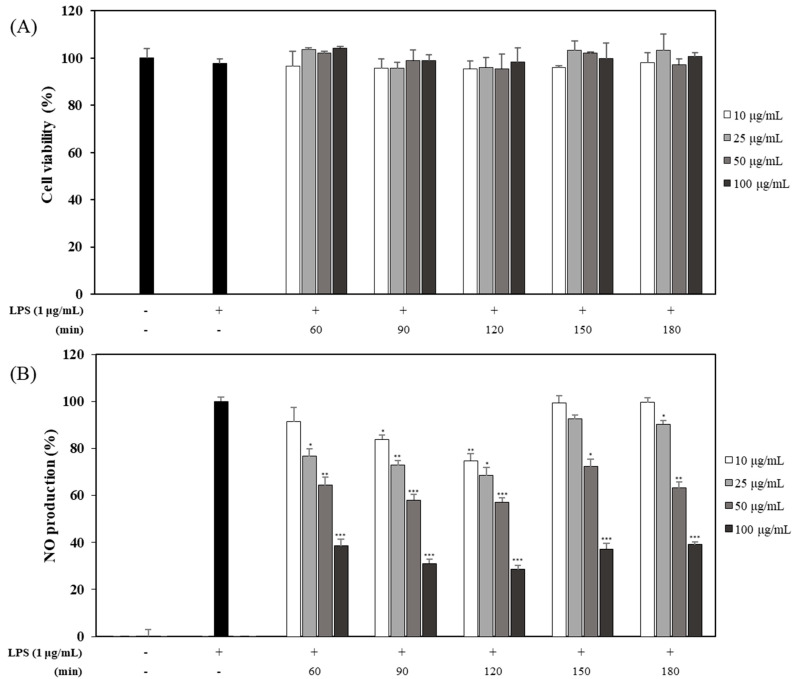
Effects of OFLEs obtained with various extraction time on cell viability and NO production. Effect of OFLEs (10, 25, 50, and 100 μg/mL) obtained with different extract time (60, 90, 120, 150, and 180 min) on LPS-stimulated cell viability (**A**) and NO production (**B**) in mouse macrophages. Cells were cultured in the presence of OFLE at various concentrations for 1 h before stimulation with 100 μg/mL LPS. At 20 h, the culture supernatant was assayed for cell viability and NO production. Values are presented as mean percentage ± SD of five samples from three different experiments compared with the corresponding LPS-induced control (100%). * *p* < 0.05, ** *p* < 0.01, *** *p* < 0.001 vs. LPS-treated group.

After the second experiment, five OFLEs obtained with a sonication time of 60, 90, 120, 150, and 180 min at different concentrations were evaluated for their effects on NO production and cell viability. Results showed that their inhibitory effects on NO production were not attributable to their effects on cell viability (Figure 3A). The OFLE obtained from leaves harvested in June and extracted with 60% EtOH for 120 min showed significantly higher inhibitory activity (69.8% inhibition) on NO production than other OFLEs at a concentration of 100 μg/mL (Figure 3B). Therefore, extracting leaves harvested in June with 60% EtOH condition for 120 min was derived as the optimal extraction method for OFLE. Such extract was named OP OFLE.

### 2.4. Effects of OP OFLE and OFLE Extracted with 60% MeOH on LPS-Induced NO Production and Cell Viability

**Figure 4:**
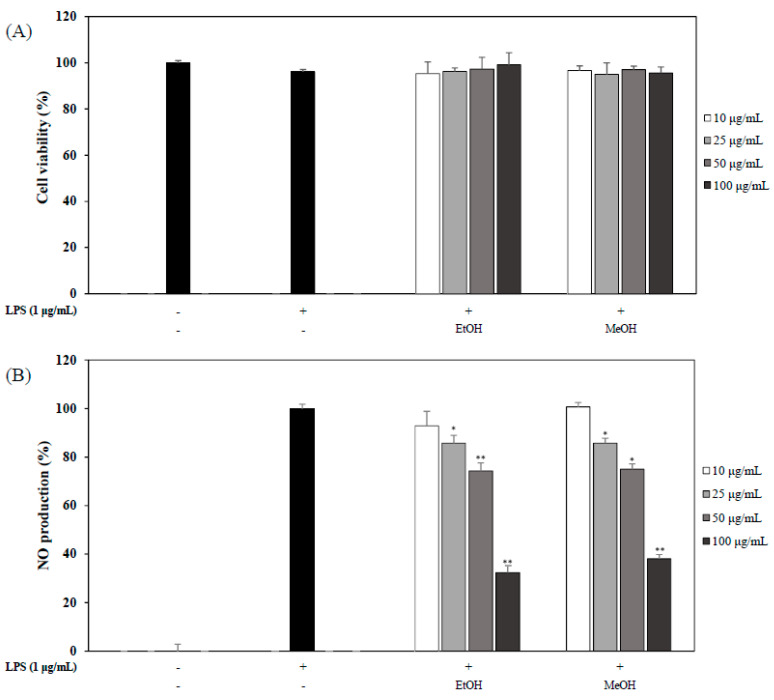
Effects of OP OFLE and MeOH OFLE on cell viability and NO production. Inhibitory effects of OP OFLE and MeOH OFLE (10, 25, 50, and 100 μg/mL) on cell viability and NO production of LPS-stimulated RAW 264.7 cells were determined. Cells were pretreated with each extract at different concentrations for 20 h. Cytotoxicity (**A**) was determined by MTT assay. Culture media were used to determine NO production (**B**) with Griess reagent. Data are represented as mean ± SD. * *p* < 0.05, ** *p* < 0.01 vs. LPS-treated group.

In the last experiment, we evaluated the effects of OP OFLE and OFLE extracted with 60% MeOH on NO production in LPS-stimulated mouse macrophages. OP OFLE and the OFLE extracted with 60% MeOH suppressed the production of NO at concentrations of 25, 50, and 100 μg/mL. In addition, they showed no cytotoxicity (Figure 4A). When these two extracts were compared, OP OFLE showed a higher inhibition effect on NO production than the methanol extract at all concentrations. In particular, OP OFLE at 100 μg/mL showed the highest NO inhibitory activity, resulting in an inhibition of 67.6% (Figure 4B).

### 2.5. DPPH and ABTS Radical Scavenging Activities of OP OFLE

**Figure 5:**
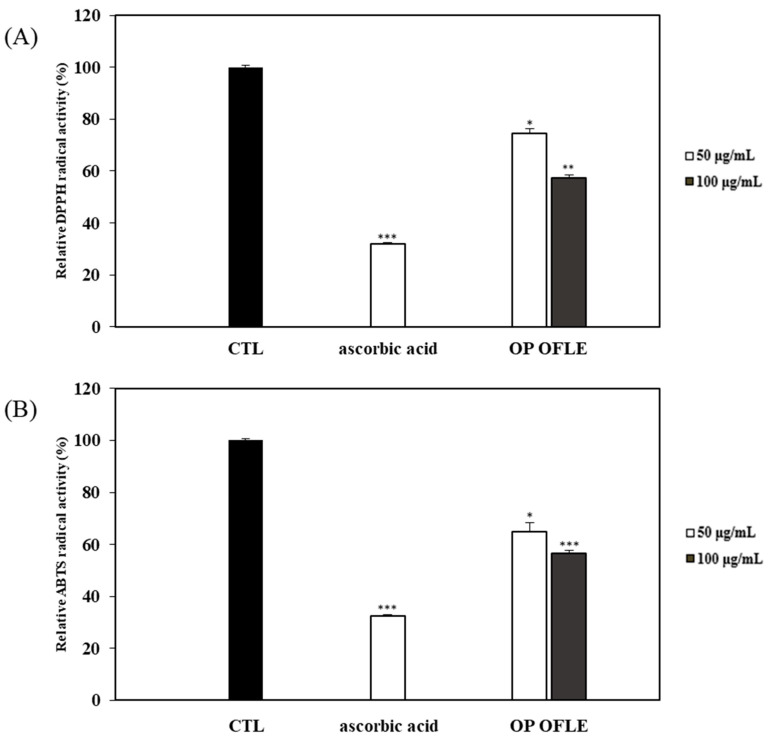
DPPH radical scavenging activity (**A**) and ABTS radical scavenging activity (**B**) of OP OFLE (50 and 100 μg/mL) are shown. Data are presented as mean ± SD (*n* = 3) of three individual experiments. * *p* < 0.05, ** *p* < 0.01, *** *p* < 0.001.

DPPH and ABTS assays were performed to investigate the antioxidant activities of OP OFLE at 50 and 100 μg/mL. In this experiment, ascorbic acid (100 μM) was used as a positive control. OP OFLE exhibited a concentration-dependent radical scavenging activity. It showed a strong antioxidant activity in both DPPH and ABTS experiments. In the DPPH assay, it showed 45.4% radical scavenging activity at 100 μg/mL. In the ABTS assay, it showed 42.6% radical scavenging activity (Figure 5).

### 2.6. Determination of Total Phenolic Content (TPC)

TPC indicates the phenolic content in a sample. Phenolic compounds present in plants have redox properties. Such properties allow them to act as antioxidants. Results were derived from a calibration curve (y = 0.0077x − 0.0224, R^2^ = 0.9925) of gallic acid (Sigma Aldrich, St. Louis, MO, USA) and expressed in gallic acid equivalents (GAE) per gram of dry OP OFLE weight. The TPC in OP OFLE was 31.5 mg GAE/g. High phenolic content is responsible for the bioactivity of OP OFLE. Therefore, this extract is expected to reveal good antioxidant and antibacterial activities.

### 2.7. Determination of Total Flavonoid Content (TFC)

TFC indicates the content of flavonoids in a sample. Results were derived from a calibration curve (y = 0.0013x − 0.0554, R^2^ = 0.9966) of quercetin (Sigma Aldrich, St. Louis, MO, USA) and expressed in quercetin equivalents (QE) per gram of dry extract weight. The TFC in OP OFLE was 18.5 mg QE/g. High flavonoid content is responsible for the bioactivity of OP OFLE. Therefore, this extract is expected to show good antioxidant and antibacterial activities.

### 2.8. Isolation of PH, Lignan Glycodise from OP OFLE

**Figure 6:**
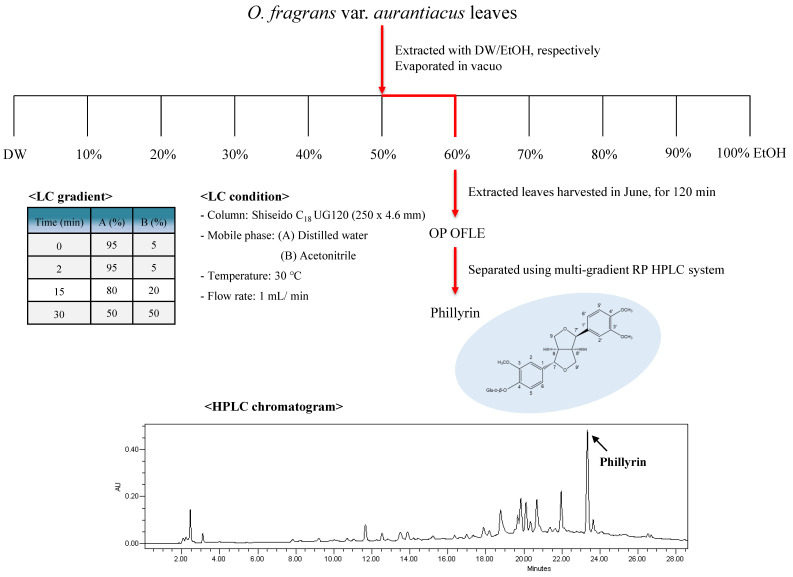
Isolation scheme and structure of PH isolation from OP OFLE.

OP OFLE was subjected to column chromatography and assigned a major compound, which was identified as lignan glycoside, PH, by analyzing its spectral data, including nuclear magnetic resonance (NMR) and liquid chromatography-mass spectroscopy (LC-MS) data with those reported studies (Figure 6) [20].

### 2.9. Effects of OP OFLE and PH Treatment on iNOS and COX-2 Expression

**Figure 7:**
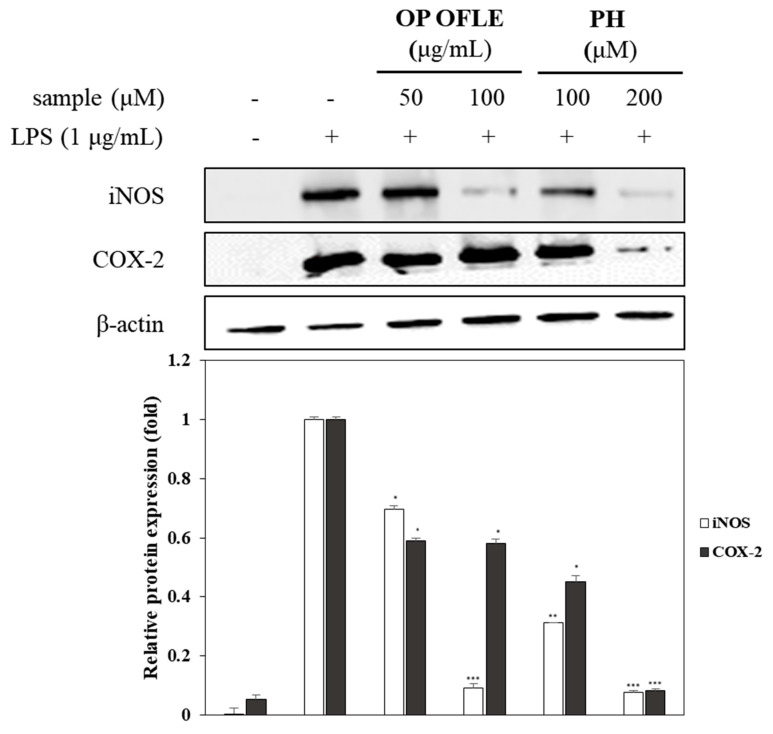
Effects of OP OFLE and PH on iNOS and COX-2 expression. RAW 264.7 cells were cultured in the presence of OP OFLE (50 and 100 μg/mL) or PH (100 and 200 μM) for 2 h and then induced with LPS (1 μg/mL) for 20 h. Levels of iNOS, COX-2, and β-actin in the LPS-stimulated cells were evaluated by Western blot analysis. Relative density was calculated as the ratio of the expression level of each protein to that of β-actin. Data are expressed as mean ± SD (*n* = 3) of three individual experiments. * *p* < 0.05, ** *p* < 0.01, *** *p* < 0.001 vs. LPS-stimulated group.

RAW 264.7 cells were used to determine the effects of OP OFLE (50, 100 μg/mL) and PH (100, 200 μM) on protein expression. As shown in Figure 7, iNOS and COX-2 protein levels were increased significantly in the group induced with LPS without any pretreatment. However, pretreatment with OP OFLE or PH resulted in dose-dependent decreases of these protein expression levels induced by LPS. Compared to the control group without any pretreatment, OP OFLE at 100 μg/mL and PH at 200 μM reduced iNOS protein expression by 91.7% and 92.5%, respectively. Additionally, we assessed effects of OP OFLE and PH on COX-2 protein through Western blot analysis. Both OP OFLE and PH inhibited its expression. Pretreatment with OP OFLE at 100 μg/mL showed the highest inhibitory effect on COX-2 protein expression, resulting in a 41.8% reduction. PH at 100 and 200 μM also attenuated the expression of COX-2 protein upregulated by LPS stimulation by 54.9% and 91.8%, respectively. The expression of LPS-stimulated COX-2 protein was inhibited by 41.8% and 91.8% after treatment with OP OFLE (100 μg/mL) and PH (200 μM), respectively (Figure 7).

### 2.10. Effects of OP OFLE and PH Treatment on NF-κB Activation and pERK1/2 Expression

**Figure 8:**
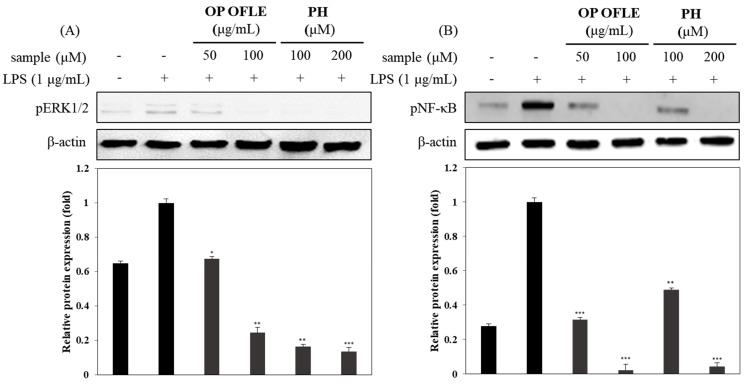
Effects of OP OFLE and PH on pERK1/2 activation and NF-κB expression. Inhibition of the OP OFLE (50 and 100 μg/mL) and PH (100 and 200 μM) on LPS-induced pERK1/2 (**A**) and NF-κB (**B**) proteins in RAW264.7 macrophages. Cells were pretreated with OP OFLE or PH at different concentrations for 2 h and then stimulated with LPS for 1 h. Expression of pERK1/2 and NF-κB proteins were detected by Western blot using specific pERK1/2 and NF-κB antibodies. β-Actin was used as a loading control. Relative density of NF-κB compared to that of β-actin was calculated. Blots shown are representatives of three independent experiments with similar results. * *p* < 0.05, ** *p* < 0.01, *** *p* < 0.001 vs. LPS-stimulated group.

It has been reported that NF-κB activation was induced by IL-1β, IL-6, TNF-α, and iNOS in RAW 264.7 cells [21]. To investigate the anti-inflammatory effects of OP OFLE and PH, Western blotting was carried out after RAW 264.7 macrophage cells were treated with OP OFLE (50, 100 μg/mL) or PH (100, 200 μM) for 2 h prior to LPS stimulation to determine their inhibitory effects on NF-κB and pERK1/2. Results show that OP OFLE and PH downregulated the expression of NF-κB and pERK1/2 in RAW 264.7 cells in a concentration-dependent manner, with OP OFLE at 100 μg/mL and PH at 200 μM showing high inhibitory effects. These results verified that OP OFLE and PH could suppress NF-κB and pERK1/2 protein expression levels (Figure 8).

### 2.11. Effects of OP OFLE and PH on Expression Levels of Pro-Inflammatory Cytokines IL-1β, IL-6, IL-8, and TNF-α

**Figure 9:**
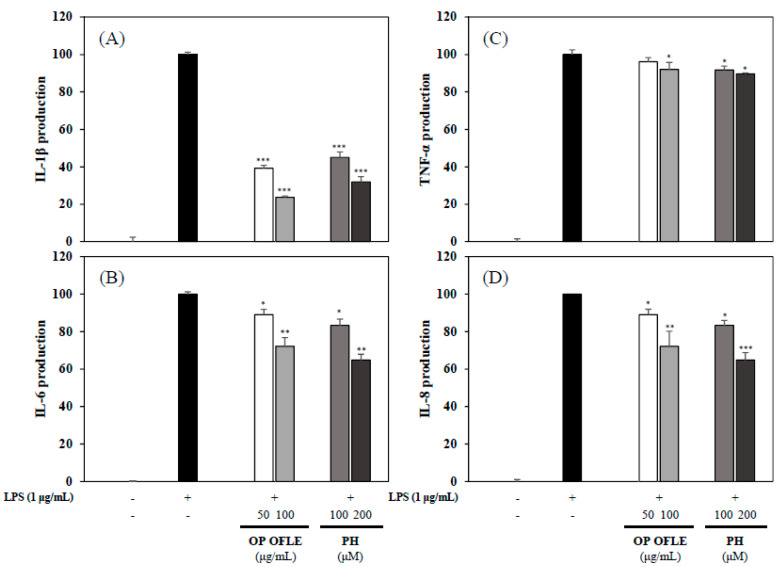
Effects of OP OFLE and PH on expression levels of IL-1β, IL-6, TNF-α, and IL-8. Inhibitory effects of OP OFLE (50 and 100 μg/mL) and PH (100 and 200 μM) on IL-1β (**A**), IL-6 (**B**), TNF-α (**C**), and IL-8 (**D**) expression levels in LPS-induced RAW 264.7 cell and HT-29 colonic epithelial cells were determined. These four proinflammatory cytokines were assessed with ELISA kits. Data are expressed as mean ± SD (*n* = 3) of three individual experiments. * *p* < 0.05; ** *p* < 0.01; and *** *p* < 0.001 vs. LPS-stimulated group.

Activated HT-29 cells and RAW 264.7 cells can produce pro-inflammatory cytokines such as IL-1β and IL-6 [22]. The inhibitory effects of OP OFLE and PH on pro-inflammatory mediators were evaluated using ELISA kits. Stimulation with LPS significantly increased levels of pro-inflammatory cytokines. However, pretreatment with OP OFLE or PH decreased levels of pro-inflammatory cytokines in a concentration-dependent manner. Particularly, IL-1β production was significantly inhibited by OP OFLE at 100 μg/mL and PH at 200 μM. In addition, OP OFLE and PH down-regulated IL-8 production in LPS-stimulated HT-29 cells. These results showed that OP OFLE and PH could suppress pro-inflammatory cytokines (Figure 9).

## 3. Discussion

Nowadays, there is an increasing recognition that inflammation is associated with the pathogenesis of diverse human diseases ranging from infection to immune-mediated disorders, diabetes, cancer, cardiovascular pathology, neurodegeneration, metabolic syndrome, and aging itself [23]. Inflammation is a normal immune process that responds to tissue damage, microbial pathogen infection, and chemical irritation. However, prolonged inflammation can cause various chronic diseases [24]. The necessity of discovering new anti-inflammatory drugs becomes important. Great effort has been made to develop drugs for treating inflammation [25].

During our search for candidates of botanical drugs to control inflammation, *O. fragrans* var. *aurantiacus* leaves have been found to possess anti-inflammatory phytochemicals. In this study, we focused on extracts of *O. fragrans* var. *aurantiacus* leaves showing the highest bioactivity. In addition, representative active ingredients were determined. To find the optimal extraction method for OFLE, different solvent ratios, harvest time of leaves, and extraction time were used. Each OFLE was then tested for its inhibitory effect on NO production. It was observed that leaves harvested in June and extracted with 60% EtOH for 120 min showed the highest inhibitory effect on NO production. We also evaluated the effect of OFLE extracted with MeOH on NO production. Results revealed that OP OFLE had a stronger inhibitory effect on NO production than MeOH OFLE. OP OFLE was then subjected to HPLC analysis to elucidate its major active constituents, yielding PH.

A previous study revealed that phenylpropanoids and lignans, such as phenylpropanoid dimers, are the main ingredients in *Osmanthus* species that possess biological activities such as antioxidant, antifungal, and antibacterial effects [26]. In particular, it has been reported that lignans including phillygenin and phillyrin from *O. fragrans* var. *aurantiacus* flowers have inhibitory activities on NO production [13]. This study evaluated the potential of OP OFLE and lignans isolated from OFLE to treat inflammation. In macrophages, LPS as a well-known endotoxin can induce the production of inflammatory mediators such as NO and prostaglandin E2 (PGE2) synthesized by iNOS and COX-2 and inflammatory cytokines such as IL-1β and TNF-α [27]. We examined the cell viability of macrophages and inhibitory effects of these extracts on NO production. Results showed that the extract suppressed NO production without showing any cytotoxicity OP OFLE and PH lignan showed strong NO production inhibitory effects. Their treatment decreased iNOS and COX-2 levels in LPS-induced RAW 264.7 cells.

MAPKs (including JNK kinase and p38MAPK) and NF-κB can activate some proinflammation cytokines [28]. NF-κB commonly regulates the promoter region of many proinflammatory cytokines. In activated macrophages, NF-κB in synergy with other transcriptional activators plays a central role in coordinating the expression of genes encoding iNOS, IL-1, IL-6, and TNF-α [29]. OP OFLE and PH decreased the phosphorylation levels of pNF-κB and pERK1/2 (Figure 8). We evaluated levels of IL-1β, IL-6, and TNF-α proteins upon treatment of cells with OP OFLE and PH and revealed that the levels of these LPS-stimulated pro-inflammatory cytokines were suppressed in macrophage and colonic epithelial cells (Figure 9). These results showed that OP OFLE and PH could potentially be considered therapeutic agents for treating inflammatory diseases by down-regulating inflammatory mediators.

Phenolic compounds including flavonoids, phenylpropanoids, and lignans are secondary metabolites of plants [30]. They have attracted increasing attention in traditional medicine for cancer prevention or treatment due to their ability to act as antioxidant agents [31]. In addition, flavonoids that are readily consumed by humans appear to exhibit anti-allergic, anti-cancer, and anti-inflammatory activities [32]. Flavonoids can exert antioxidant activities by donating hydrogen atoms to free radicals to suppress the generation of free radicals excessively produced by oxidative stress in vivo [33]. Lignans exhibit numerous biological activities, including antiproliferative and antioxidant effects. In addition, they have protective functions against cardiovascular diseases and other degenerative pathologies associated with oxidative stress [34]. As a result of evaluating TPC and TFC, *O. fragrans* var. *aurantiacus* leaves were found to contain high levels of phenolic compounds and flavonoids. *O. fragrans* var. *aurantiacus* leaves possess high amounts of phenolic compounds, including lignan and flavonoids with potent antioxidant activities. Therefore, they have disease fighting properties and provide various defenses. Additionally, the antioxidant effects of OP OFLE were assessed with DPPH and ABTS assays by measuring radical scavenging capacity [35]. Results of DPPH and ABTS assays showed that OP OFLE at 100 μg/mL concentration exhibited high radical scavenging activities (DPPH and ATBS: 45.4% and 42.6%, respectively).

In summary, we determined the optimal extraction method by evaluating the anti-inflammatory effects of OFLEs on NO production in LPS-induced macrophages. OP OFLEs exhibited potent DPPH and ABTS radical scavenging activities and inhibited NO production. Compounds **1**–**3** isolated from leaves of *O. fragrans* var. *aurantiacus* showed no cytotoxicity. OP OFLE and PH isolated as a major compound from it downregulated the iNOS, COX-2, pNF-κB, and p-ERK levels. In addition, levels of pro-inflammatory cytokines such as IL-1β, IL-6, IL-8, and TNF-α were decreased by treatment with OP OFLE and PH. We found that the anti-inflammatory activity of PH isolated from *O. fragrans* var. *aurantiacus* leaves is due to different mechanisms of action. On the basis of previous studies and our results, OP OFLE could be used as a potential antioxidant with anti-inflammatory properties and applied to cosmetics, health functional foods, and pharmaceutical compositions

## 4. Materials and Methods

### 4.1. Plant Materials

Leaves of *O. fragrans* var. *aurantiacus* were collected from Seoul National University Forests, Gwangyang, Korea in March, June, and September of 2018. A voucher specimen (SCNUP 21) was deposited at the laboratory of Pharmacognosy, College of Pharmacy, Sunchon National University, Suncheon, Jeonnam, Korea.

### 4.2. Preparation of Extracts

To prepare ethanol extracts, 1 g of ground *O. fragrans* var. *aurantiacus* leaves was mixed with 10 mL of 0, 10, 20, 30, 40, 50, 60, 70, 80, 90, or 100% EtOH and extracted by sonication at room temperature (RT) (2 h × 2 cycles). Extracts were filtered through No. 2 Whatman filter paper (Whatman, Pleasanton, CA, USA) and evaporated in vacuo at 39 °C using a rotary evaporator (Eyela; Tokyo, Japan). Finally, concentrated extracts were kept in dark at 4 °C. After the first experiment, three candidates (extracts with 60, 70, and 80% EtOH) were selected. Leaves were then extracted by sonication according to their harvest time (March, June, and September). After the second experiment, the harvest time and the extraction solvent mixture ratio condition (June, 60% EtOH) were selected and sonication extraction time was performed at five conditions (60, 90, 120, 150, 180 min). After the first, second, and third experiments, the optimum extraction condition for leaves of *O. fragrans* var. *aurantiacus* was derived as extraction of leaves harvested in June with 60% EtOH for 120 min. In the final experiment, the OP OFLE was compared with the extract obtained with 60% methanol.

### 4.3. Extraction and Isolation

The OP OFLE was additionally obtained by extraction 10 g of leaves and then dissolved with 100% MeOH and separated by column chromatography. Compounds (3.4 mg, *t_R_* 23.38 min) were obtained after isolation using a multi-gradient reverse phase (RP) HPLC system (Shiseido, C_18,_ 250 × 10 mm, H_2_O:CH_3_CN = 95:5 → 30:70, 1 mL/min) and were then subjected to proton NMR (^1^H-NMR), carbon NMR (^13^C-NMR), and LC-MS analyses.

Phillyrin (PH): C_27_H_34_O_11_; white amorphous powder; ^1^H-NMR (400 MHz, C_5_D_5_N): *δ*_H_ 6.85–7.05 (2H, m, H-2, 2′), 6.85–7.05 (2H, m, H-5, 5′), 6.85–7.05 (2H, m, H-6, 6′), 4.87 (1H, d, *J* = 6.4 Hz, Glc-1), 4.10 (1H, d, *J* = 9.6 Hz, H-9α), 3.75 (3H, s, 3-OCH_3_), 3.75 (3H, s, 3′-OCH_3_), 3.75 (3H, s, 4-OCH_3_), 3.36 (2H, d, *J* = 6.8 Hz, H-7, 7′), 3.05–3.71 (4H, m, H-8′, 9β, 9′), 3.05–3.71 (5H, m, Glc-2, 3, 4, 5, 6), 2.84 (1H, m, H-8); ^13^C NMR (100 MHz, C_5_D_5_N): *δ*_C_ 148.9 (C-3, 3′), 145.9 (C-4, 4′), 135.3 (C-1, 1′), 118.2 (C-6, 6′), 115.2 (C-5, 5′), 110.4 (C-2, 2′), 100.1 (Glc-1), 86.7 (C-7, 7′), 77.0 (Glc-5), 76.9 (Glc-3), 73.2 (Glc-2), 70.3 (C-9, 9′), 69.7 (Glc-4), 60.7 (Glc-6), 55.7 (3-OCH_3_), 55.3 (3′-OCH_3_), 55.5 (4-OCH_3_), 54.0 (C-8, 8′); ESIMS *m*/*z* 535.32 [M + H]^+^.

### 4.4. Cell Culture

Human colon epithelial cells, HT-29 cells, and mouse macrophages, RAW 264.7 cells, were purchased from Korean Cell bank (Seoul, Korea). These cell lines were cultured in an incubator at 37 °C with a humidified atmosphere and 4.5% CO_2_. These cell lines were grown in Dulbecco’s modified Eagle’s medium (DMEM) (Hyclone, Logan, UT, USA) supplemented with 100 μg/ mL streptomycin solution (Hyclone, Logan, UT, USA), 10% fetal bovine serum (FBS) (HyClone, Logan, UT, USA), and 100 IU/mL penicillin.

### 4.5. Cell Viability Assay

RAW 264.7 cells were plated in a 96-well plate at a density of 1 × 10^5^ cells/well in DMEM containing 10% FBS and 1% penicillin for 24 h. Cells were treated with different concentrations of samples for 1 h and then stimulated with LPS at 1 μg/mL for 20 h. Cell viability was measured by 3-(4,5-dimethyl-2-thiazolyl)-2,5-diphenyl tetrazolium bromide (MTT) assay. Cultured cells were incubated with MTT (0.05 mg/mL) at 37 °C for 4 h. After removing the media, 100 μL DMSO was added. After shaking for 5 min, absorbance was measured at 570 nm using a microplate reader (Bio Tek Instruments, Winooski, VT, USA).

### 4.6. Measurement of NO Production

The supernatant of NO culture produced by RAW 264.7 cells were assessed by Griess reagent. The RAW 264.7 cells were planted at 1 × 10^5^ cells/well density in a 96-well plate and cultured in an incubator for 24 h. Cells were then treated with samples at different concentrations for 1 h and then stimulated with LPS for 20 h. The supernatant was then harvested and the same amount of the Griess reagent was added to the supernatant. The mixture was reacted in dark conditions at RT for 15 min and the absorbance was then measured at a wavelength of 550 nm using a microplate reader.

### 4.7. Measurement of Cytokine Production

Levels of IL-1β, IL-6, and TNF-α in RAW 264.7 cells and the level of IL-8 in HT-29 cells were measured using ELISA kits (BD OptEIA™, San Diego, CA, USA) according to the manufacturer’s instructions.

### 4.8. Western Blot Analysis

RAW 264.7 cells were seeded in 6-well plates at a density of 1 × 10^5^ cells/well. After treatment with OP OFLE (50, 100 μg/mL) and PH (100, 200 μM), cells were stimulated with LPS. Subsequently, protein was extracted using proprep solution (iNtRON, Biotechnology, Daejeon, Korea) for each treatment group. Protein concentrations were quantified using BCA solution through Bradford assay. The same amount of protein was separated by electrophoresis using 10% sodium dodecyl sulfate-poly acrylamide gel. Separated proteins were transferred to PVDF membranes using a transfer device (Thermo Fisher Scientific, Waltham, MA, USA). Membranes were then blocked with 5% skim milk power in platin buffer [4 M NaCl and 1 M Tris-HCl (PH 7.5)] for one hour at RT. Primary antibodies against iNOS, COX-2, NF-κB_,_ ERK1/2, and pERK1/2 were incubated with the membrane for 24 h at 4 °C. Membranes were then washed with wash buffer (4 M NaCl, Tween-20, 1 M Tris-HCl (PH 7.5)) in DW and then incubated with a second antibody (1:2000 dilution) at RT for 2 h. Protein signals were detected with a bio image system (Microchemi 4.2 Chemilumineszenz-System, Neve Yamin, Israel) using ECL solution (Thermo Fisher Scientific, Waltham, MA, USA) [36].

### 4.9. Total Phenolic Content

Total phenolics content (TPC) of each extract was determined using the Folin–Denis method [37]. Briefly, 160 μL of sample was dissolved in EtOH (5, 10 mg/mL) and mixed thoroughly with 160 μL of Folin–Denis reagent (Sigma-Aldrich Co., St. Louis, MO, USA) for 3 min at RT. After adding 160 μL of 10% (*w*/*v*) sodium carbonate, the mixture was allowed to stand at RT for a further 60 min in the dark. After centrifugation at 10,000 rpm for 10 min, the supernatant (100 μL) was collected and its absorbance was measured at 700 nm. A standard curve was prepared using gallic acid (Sigma-Aldrich, St. Louis, MO, USA) with a concentration range from 0 to 500 μg/mL. Total phenolic content was expressed as mg gallic acid equivalents (GAE)/g of sample.

### 4.10. Total Flavonoid Content

Total flavonoid content of sample was determined by the Moreno method [38]. Briefly, 10 μL of each extract (5, 10 mg/mL EtOH) was mixed with 4 μL of 10% aluminum nitrate (Sigma-Aldrich Co.), 4 μL of 1 M potassium acetate (Sigma-Aldrich Co.), and 82 μL of MeOH. The mixture was allowed to stand at RT for a further 40 min in the dark. Its absorbance was then measured at 415 nm. A standard curve was prepared using quercetin (Sigma-Aldrich Co.) with a concentration range from 0 to 500 μg/mL. Total flavonoid content was expressed as mg quercetin equivalents (QE)/g of sample.

### 4.11. DPPH Radical Scavenging Activity Assay

The electron donating ability (EDA) was measured by modifying Amarowicz’s method [39] to determine DPPH radical inhibitory activity. Briefly, 0.1 mL of each sample solution (dissolved in EtOH) was mixed with 0.1 mL of 0.2 mM 2,2-diphenyl-1-picrylhydrazyl (DPPH, Sigma-Aldrich Co.) and allowed to stand at RT for 30 min. Its absorbance was then measured at 517 nm. The electron donating ability was expressed as the absorbance reduction rate of the sample solution added group compared to the no addition group using the following formula: DPPH radical scavenging activity (%) = (1 − absorbance of sample addition group/absorbance of no addition group) × 100. As a positive control, 2,6-di-tert-butyl-4-methylphenol (BHT, Sigma-Aldrich Co.), a commercially available antioxidant, was used [40].

### 4.12. ABTS Radical Scavenging Activity Assay

ABTS radical inhibitory activity was measured by mixing 0.1 mL of each sample solution (dissolved in EtOH) and 0.1 mL of ABTS solution (7.4 mM 2,2-azino-bis(3-rthylbenzthiazoline-6-sulfonic acid); ABTS) mixed with 2.6 mM potassium persulfate). After incubating at RT for 6 min, the absorbance of the mixture was measured at 734 nm. The electron donating ability was expressed as the absorbance reduction rate of the sample solution added group compared to the no addition group [40].

### 4.13. Statistical Analysis

All data are expressed as means ± standard deviation (SD) of at least three independent experiments. Comparison of data was achieved using Duncan’s multiple range tests with the software package SPSS statistics version 20. Statistical significance was indicated by asterisks (* *p* < 0.05; ** *p* < 0.01; and *** *p* < 0.001).

## Data Availability

Not applicable.

## References

[1] Krishnamoorthy S., Honn K.V. (2006). Inflammation and Disease Progression. Cancer Metastasis Rev..

[2] Willerson J.T., Ridker P.M. (2004). Inflammation as a Cardiovascular Risk Factor. Circulation.

[3] Chen L., Deng H., Cui H., Fang J., Zuo Z., Deng J., Li Y., Wang X., Zhao L. (2017). Inflammatory Responses and Inflammation-Associated Diseases in Organs. Oncotarget.

[4] Haddad P.S., Azar G.A., Groom S., Boivin M. (2005). Natural Health Products, Modulation of Immune Function and Prevention of Chronic Diseases. Evid.-Based Complement. Altern. Med..

[5] Chun S., Jee S.Y., Lee S.G., Park S.J., Lee J.R., Kim S.C. (2007). Anti-Inflammatory Activity of the Methanol Extract of Moutan Cortex in LPS-Activated Raw264.7 Cells. Evid.-Based Complement. Altern. Med..

[6] Jung Y.C., Kim M.E., Yoon J.H., Park P.R., Youn H., Lee H., Lee J.S. (2014). Anti-Inflammatory Effects of Galangin on Lipopolysaccharide-Activated Macrophages Via ERK and NF-κB Pathway Regulation. Immunopharmacol. Immunotoxicol..

[7] Xie Q., Nathan C. (1994). The high-output Nitric Oxide Pathway: Role and Regulation. J. Leukoc. Biol..

[8] Moro C., Palacios I., Lozano M., D’Arrigo M., Guillamón E., Villares A., Martínez J.A., García-Lafuente A. (2012). Anti-Inflammatory Activity of Methanolic Extracts from Edible Mushrooms in LPS Activated RAW 264.7 Macrophages. Food Chem..

[9] Chen X., Zhang J., Liu J., Yu B. (2008). Biotransformation of p-, m-, and o-Hydroxybenzoic Acids by Panax Ginseng Hairy Root Cultures. J. Mol. Catal. B.

[10] Zhou H.Y., Shin E.M., Guo L.Y., Youn U.J., Bae K., Kang S.S., Zou L.B., Kim Y.S. (2008). Anti-Inflammatory Activity of 4-Methoxyhonokiol is a Function of the Inhibition of iNOS and COX-2 Expression in RAW 264.7 Macrophages Via NF-κB, JNK and p38 MAPK Inactivation. Eur. J. Pharmacol..

[11] Kim S., Shin T. (2009). Anti-Inflammatory Effect of Leaves of *Eriobotrya japonica* Correlating with Attenuation of p38 MAPK, ERK, and NF-κB Activation in Mast Cells. Toxicol. Vitro.

[12] Bai A.P., Ouyang Q., Zhang W., Wang C.H., Li S.F. (2004). Probiotics Inhibit TNF-Alpha-Induced Interleukin-8 Secretion of HT29 Cells. World J. Gastroenterol..

[13] Lee D., Lee S., Bang M., Park H., Lee T., Kim Y., Kim J., Baek N. (2011). Lignans from the Flowers of *Osmanthus Fragrans* var. *aurantiacus* and their Inhibition Effect on NO Production. Arch. Pharm. Res..

[14] Lee K.J., Baek D., Lee G., Cho G., So Y., Lee J., Ma K., Chung J., Hyun D.Y. (2020). Phytochemicals and Antioxidant Activity of Korean Black Soybean (*Glycine Max* L.) Landraces. Antioxidants.

[15] Lee C.Y., Sharma A., Semenya J., Anamoah C., Chapman K.N., Barone V. (2020). Computational Study of Ortho-Substituent Effects on Antioxidant Activities of Phenolic Dendritic Antioxidants. Antioxidants.

[16] Wang L., Oh J.Y., Hwang J., Ko J.Y., Jeon Y., Ryu B. (2019). In Vitro and in Vivo Antioxidant Activities of Polysaccharides Isolated from Celluclast-Assisted Extract of an Edible Brown Seaweed, *Sargassum fulvellum*. Antioxidants.

[17] Machida K., Yamauchi M., Kurashina E., Kikuchi M. (2010). Four New Lignan Glycosides from *Osmanthus fragrans* Lour. var. *aurantiacus* Makino. Helv. Chim. Acta.

[18] Jeong D.E., Shim S., Lee M. (2020). Anti-Inflammatory Activity of Phenylpropyl Triterpenoids from *Osmanthus fragrans* var. *aurantiacus* Leaves. Int. Immunopharmacol..

[19] Hou P., Regenstein J. (2004). Optimization of Extraction Conditions for Pollock Skin Gelatin. J. Food Sci..

[20] Kwak J.H., Kang M.W., Roh J.H., Choi S.U., Zee O.P. (2009). Cytotoxic Phenolic Compounds from *Chionanthus retusus*. Arch. Pharm. Res..

[21] Schumacher B., Scholle S., Hölzl J., Khudeir N., Hess S., Müller C.E. (2002). Lignans Isolated from Valerian: Identification and Characterization of a New Olivil Derivative with Partial Agonistic Activity at A1 Adenosine Receptors. J. Nat. Prod..

[22] Shim S., Lee S., Lee M. (2018). Biflavonoids Isolated from *Selaginella tamariscina* and their Anti-Inflammatory Activities via ERK 1/2 Signaling. Molecules.

[23] Rea I.M., Gibson D.S., McGilligan V., McNerlan S.E., Alexander H.D., Ross O.A. (2018). Age and age-related diseases: Role of inflammation triggers and cytokines. Front. Immunol..

[24] Pan M.H., Lai C.S., Ho C.T. (2010). Anti-inflammatory activity of natural dietary flavonoids. Food Funct..

[25] Andrade L.N., De Sousa D.P. (2013). A review on anti-inflammatory activity of monoterpenes. Molecules.

[26] Zhou F., Peng J., Zhao Y., Huang W., Jiang Y., Li M., Wu X., Lu B. (2017). Varietal Classification and Antioxidant Activity Prediction of *Osmanthus fragrans* Lour. Flowers using UPLC–PDA/QTOF–MS and Multivariable Analysis. Food Chem..

[27] Sakanaka S., Kim M., Taniguchi M., Yamamoto T. (1989). Antibacterial Substances in Japanese Green Tea Extract Against *Streptococcus mutans*, a Cariogenic Bacterium. Agric. Biol. Chem..

[28] Zhang T., Wang M., Yang L., Jiang J., Zhao J., Zhu W. (2015). Flavonoid Glycosides from Rubus Chingii Hu Fruits Display Anti-Inflammatory Activity through Suppressing MAPKs Activation in Macrophages. J. Funct. Foods.

[29] Qiao H., Zhang X., Zhu C., Dong L., Wang L., Zhang X., Xing Y., Wang C., Ji Y., Cao X. (2012). Luteolin Downregulates TLR4, TLR5, NF-κB and p-p38MAPK Expression, Upregulates the p-ERK Expression, and Protects Rat Brains Against Focal Ischemia. Brain Res..

[30] Park J.C., Yoo H., Kim C.E., Shim S.Y., Lee M. (2017). Hispidulin-7-O-Neohesperidoside from *Cirsium japonicum* var. *Ussuriense* Attenuates the Production of Inflammatory Mediators in LPS-Induced Raw 264.7 Cells and HT-29 Cells. Pharmacogn. Mag..

[31] Johari M.A., Khong H.Y. (2019). Total Phenolic Content and Antioxidant and Antibacterial Activities of *Pereskia bleo*. Adv. Pharmacol. Sci..

[32] Fattahi S., Zabihi E., Abedian Z., Pourbagher R., Motevalizadeh Ardekani A., Mostafazadeh A., Akhavan-Niaki H. (2014). Total Phenolic and Flavonoid Contents of Aqueous Extract of Stinging Nettle and in Vitro Antiproliferative Effect on Hela and BT-474 Cell Lines. Int. J. Mol. Cell. Med..

[33] Rebaya A., Belghith S.I., Baghdikian B., Leddet V.M., Mabrouki F., Olivier E., Cherif J., Ayadi M.T. (2014). Total Phenolic, Total Flavonoid, Tannin Content, and Antioxidant Capacity of *Halimium halimifolium* (Cistaceae). J. Appl. Pharm. Sci..

[34] Harborne J., Green P. (1980). A Chemotaxonomic Survey of Flavonoids in Leaves of the Oleaceae. Bot. J. Linn. Soc..

[35] Kedare S.B., Singh R.P. (2011). Genesis and development of DPPH method of antioxidant assay. J. Food Sci. Tehcnol..

[36] Song H.Y., Jo A., Shin J., Lim E.H., Lee Y.E., Jeong D.E., Lee M. (2019). Anti-Inflammatory Activities of Isogosferol, a Furanocoumarin Isolated from *Citrus junos* Seed Shells through Bioactivity-Guided Fractionation. Molecules.

[37] Folin O., Denis W. (1912). On Phosphotungstic-Phosphomolybdic Compounds as Color Reagents. J. Biol. Chem..

[38] Moreno M.I.N., Isla M.I., Sampietro A.R., Vattuone M.A. (2000). Comparison of the Free Radical-Scavenging Activity of Propolis from several Regions of Argentina. J. Ethnopharmacol..

[39] Amarowicz R., Naczk M., Shahidi F. (2000). Antioxidant Activity of Crude Tannins of Canola and Rapeseed Hulls. J. Am. Oil Chem. Soc..

[40] Shim S., Lee Y.E., Song H.Y., Lee M. (2020). p-Hydroxybenzoic Acid β-D-Glucosyl Ester and Cimidahurinine with Antimelanogenesis and Antioxidant Effects from *Pyracantha angustifolia* Via Bioactivity-Guided Fractionation. Antioxidants.

